# The driving force of prophages and CRISPR-Cas system in the evolution of *Cronobacter sakazakii*

**DOI:** 10.1038/srep40206

**Published:** 2017-01-06

**Authors:** Haiyan Zeng, Jumei Zhang, Chensi Li, Tengfei Xie, Na Ling, Qingping Wu, Yingwang Ye

**Affiliations:** 1 State Key Laboratory of Applied Microbiology, South China, Guangdong Provincial Key Laboratory of Microbiology Culture Collection and Application, Guangdong Open Laboratory of Applied Microbiology, Guangdong Institute of Microbiology, Guangzhou 510070, China

## Abstract

*Cronobacter sakazakii* is an important foodborne pathogens causing rare but life-threatening diseases in neonates and infants. CRISPR-Cas system is a new prokaryotic defense system that provides adaptive immunity against phages, latter play an vital role on the evolution and pathogenicity of host bacteria. In this study, we found that genome sizes of *C. sakazakii* strains had a significant positive correlation with total genome sizes of prophages. Prophages contributed to 16.57% of the genetic diversity (pan genome) of *C. sakazakii*, some of which maybe the potential virulence factors. Subtype I-E CRISPR-Cas system and five types of CRISPR arrays were found in the conserved site of *C. sakazakii* strains. CRISPR1 and CRISPR2 loci with high variable spacers were active and showed potential protection against phage attacks. The number of spacers from two active CRISPR loci in clinical strains was significant less than that of foodborne strains, it maybe a reason why clinical strains were found to have more prophages than foodborne strains. The frequently gain/loss of prophages and spacers in CRISPR loci is likely to drive the quick evolution of *C. sakazakii*. Our study provides a new insight into the co-evolution of phages and *C. sakazakii*.

*Cronobacter sakazakii* is an important foodborne pathogen associated with outbreaks of life-threatening necrotizing enterocolitis, meningitis, and sepsis in neonates and infants^1^,^2^. Although the incidence is low, the fatality rates of these diseases, caused by *C. sakazakii* infection, range between 40 to 80% and survivors are often left with severe neurological and developmental disorders^3^,^4^. Infections caused by *Cronobacter spp*. have been epidemiologically linked to the consumption of contaminated powdered infant form^4^. However, virulence genes and mechanism of pathogenicity of *C. sakazakii* remain unclear^5^.

As the most abundant biological form on the planet, phages constitute major players shaping bacterial communities in most ecosystems^6^, and they are closely associated with the virulence and evolution of several important bacterial pathogens^7^. External DNA acquired by horizontal gene transfer (HGT) is the main driving force in the evolution of bacterial genomes. Integrated phages (prophages), which represent a sizable fraction of bacterial chromosomes, are major contributors to differences among individuals within a bacterial species^8^. Although acquisition of phage DNA can increase the fitness of host bacteria under certain environmental conditions, the replication and maintenance of these nucleotide sequences could be a burden^9^. Overall, predation by phages presents a serious challenge to bacterial survival; hence, bacteria have evolved numerous mechanisms to resist phage infection^9^.

CRISPR-Cas system, comprised of clustered regularly interspaced short palindromic repeats (CRISPR) along with their associated (Cas) proteins, protects prokaryotic organisms from viral predation and invading nucleic acids^10^. A CRISPR array is composed of a cluster of identical short repeats separated by short variable DNA sequences of similar length (called ‘spacers’). Such sequences derived from phage or plasmid genomes that match the sequences of CRISPR spacers are called protospacers^11^. CRISPR–Cas system encompasses three distinct mechanistic stages: adaptation, expression and interference^12^,^13^,^14^,^15^,^16^. The adaptation stage involves the incorporation of new spacers deriving from foreign DNA (‘protospacers’) into the CRISPR array. These spacers preserve the sequence memory for a targeted defense against subsequent invasions by the corresponding phages or plasmid. The expression stage includes the transcription of CRISPR sequences and subsequent processing to produce CRISPR RNAs (crRNAs). During the interference stage, crRNAs, aided by Cas proteins, function as guides to specifically target and cleave the nucleic acids of cognate phages or plasmids^10^,^11^,^15^,^16^,^17^. CRISPR-Cas system is found in 48% of Eubacteria and 95% of Archaea and has been divided into two classes, five types, and 16 subtypes, based on the signature protein families and features of the architecture of *cas* loci^10^. Recent studies have reported that CRISPR-Cas systems could also be used for non-defense roles, such as regulation of collective behavior and pathogenicity^11^.

Previous studies have found that prophages and subtype I-E CRISPR-Cas system exist in the genomes of *C. sakazakii* strains^18^,^19^,^20^,^21^. However, the characteristic of integrated prophages and CRISPR-Cas system, and their impact on the evolution of *C. sakazakii* have not been reported. In this study, we combined 17 new whole genome sequences of *C. sakazakii* strains representing the major sequence types in China obtained from this study, with public whole genome sequences to explore the contribution of prophages and CRISPR-Cas system to the evolution of *C. sakazakii.* It is meaningful for further understanding of genetic diversity of *C. sakazakii* and provides a new insight to explore their pathogenicity in the future.

## Results and Discussion

### Impact of prophages on genetic diversity and pathogenicity of *C. sakazakii*

**Figure 1:**
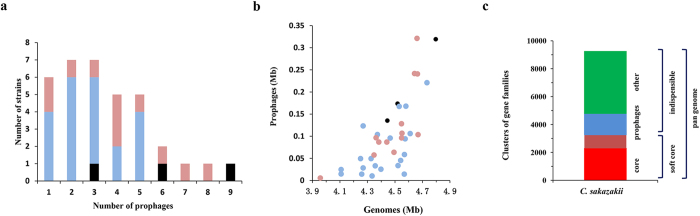
Impact of prophages on genetic diversity of *Cronobacter sakazakii*. (**A**) Frequencies of integrated prophages in the genomes of *C. sakazakii* strains. The strains isolated from clinical sample, food and environmental sample were colored with light red, light blue and black, respectively. (**B**) Correlation between genomic sizes of *C. sakazakii* and prophages. There was a significant positive correlation between the genome sizes of *C. sakazakii* strains and integrated prophages (Spearman’s *ρ* = 0.74, *P* < 10^−4^). The strains were colored the same as in Fig. 1A. (**C**) Contribution of prophage genes to genomic composition of *C. sakazakii*. The core genome corresponds to genes present in all strains, whereas the soft-core genome indicates genes present in more than 95% of the strains. The indispensable genome was split into two categories: prophages and other genes.

Prophages or prophage-derived elements were detected in all *C. sakazakii* strains. The average GC content of prophages (52.71%; ranging from 46.76% to 58.30%) was lower than that of *C. sakazakii* strains (57.04%; ranging from 56.62% to 57.70%). As shown in Fig. 1a, the number of integrated prophages in *C. sakazakii* strains ranged from 1 to 9, 83.78% of strains have less than 6 prophages. For there were only three environmental strains, we will focus on the comparison between clinical strains (n = 12) and foodborne strains (n = 21) in all of our analyses. Clinical strains had more prophages (mean: 4.15) than foodborne strains (mean: 2.81) with statistical significance (p < 0.05). The total genome sizes of prophages were 5.5–321.2 kb, contributing to 0.14–6.89% of *C. sakazakii* genome. As shown in Fig. 1b, the genome size of *C. sakazakii* increased with increase in total genome size of prophages, indicating a significant positive correlation. It implied that prophage is an important factor associated with the genome size of *C. sakazakii*. For the number of prophages in clinical strains was more than that of foodborne strains, the total genome size of prophages in clinical strains (mean: 125.79Kb) were also larger than that of foodborne strains (mean: 70.50Kb) with statistical significance (p < 0.05). Prophages can contribute important biological properties to their bacterial hosts, providing bacterial pathogens with virulence factors and accessory genes for the host fitness etc^7^,^8^. More prophages and prophages related genes may be benefit for the survival and pathogenicity of clinical strains.

Pan genome analysis of *C. sakazakii* showed that there were 9245 gene families, including 2294 core genes, 3258 soft core genes (found in 95% of the strains, includes core genes), and 5987 indispensable genes. As shown in Fig. 1c, prophage genes contribute up to 16.54% (1529/9245) of pan genome and 25.54% (1529/5987) of indispensable genome. Moreover, 64.29% (983/1529) of the prophage genes were present in one or two *C. sakazakii* strains, suggesting their rapid loss upon acquisition, contributing to the open pan genome of this species. Some of the cargo genes carried by prophages, remaining in the chromosome of *C. sakazakii*, might be potential virulence factors. For example, a prophage in *C. sakazakii* strain cro360A2 contains genes encoding a protein homologous to the heat-labile enterotoxin A peptide and a prophage in strain BAA894 encodes a protein similar to the *eae*-like adhesion protein in *Escherichia coli*^20^. Both were recognized as virulence factors in enteropathogenic *E. coli*^22^,^23^. The results implied that prophages played an important role in genetic diversity and pathogenicity of *C. sakazakii*.

### The identification of CRISPR-Cas systems in *C. sakazakii*

**Figure 2:**
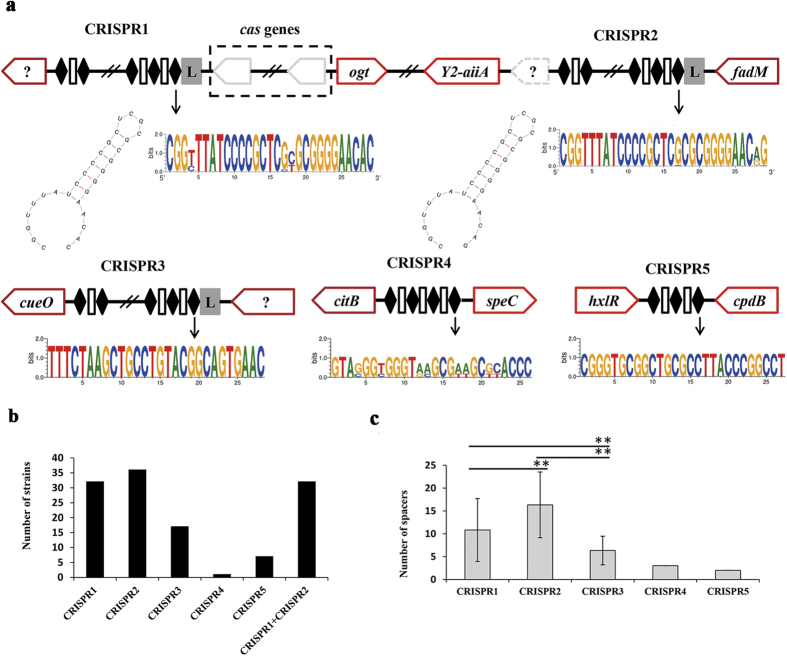
Genomic architecture of CRISPR-Cas systems in *Cronobacter sakazakii* and potential protection provided by these systems against phages. (**A**) Location of five CRISPR arrays in *C. sakazakii*. The order and orientation of genes and CRISPR arrays were drawn based on the genome of ATCCBAA894 strain. CRISPR1 was located between *cas* gene and a hypothetical gene. CRISPR2 was located between *Y2-aiiA* gene and *fadM* gene, less than 20 kb downstream of *cas* genes. In some cases, a hypothetical gene (gray dotted line) was found inserted between the *Y2-aiiA* and CRISPR2. The Weblogo and RNA secondary structure of consensus repeat sequences in CRISPR1 and CRISPR2 are indicated (below). CRISPR3, CRISPR4 and CRISPR5 were located between *cueO* gene and hypothetical gene, *citB* gene and *speC* gene, *hxlR* gene and *cpdB* gene, respectively. Hypothetical genes were indicated by a question mark. Genes with red, dark red, and gray edges represent the core, soft-core, and indispensable genes, respectively. L: AT-rich leader sequence region; Black diamond: repeat; Square: spacer. (**B**) Frequencies of five type of CRISPR arrays in *Cronobacter sakazakii* strains. (**C**) Number of spacers from five CRISPR arrays in *Cronobacter sakazakii* strains. CRISPR4 and CRISPR5 had a stable number of three and two spacers, respectively. The difference between the number of spacers in CRISPR1, CRISPR2, and CRISPR3 had statistically significance. **P < 0.001 (t-test, two tail).

CRISPR arrays were found in 97.30% (36/37) of the strains. Moreover, 94.59% (35/37) of the strains had more than one CRISPR arrays. Ogrodzki *et al*. found 12 different CRISPR spacer arrays in four major sequence types (STs) according to the composition of spacers^21^. We also found a lot of different CRISPR spacer arrays in this study, moreover, all of them were located in conserved region of *C. sakazakii* genomes. As shown in Fig. 2a, five types of CRISPR arrays were detected in *C. sakazakii* according to the conserved location. Only CRISPR1, CRISPR2 and CRISPR3 had AT-rich leader sequences. CRISPR1 and CRISPR2 were simultaneously found in 86.49% (32/37) of the *C. sakazakii* strains. However, CRISPR3, CRISPR4, and CRISPR5 were only detected in a few strains (Fig. 2b). The spacers of CRISPR1, CRISPR2, and CRISPR3 had a high variability, implying their frequent gain and loss in *C. sakazakii* (Fig. 2c). Moreover, CRISPR2 had the largest average number of spacers. Only one *cas* gene locus, which was downstream of CRISPR1 and less than 20 kb upstream of CRISPR2, was detected in the *C. sakazakii* strains (Fig. 2a). The leader sequence and *cas* genes are two determinants strictly associated with active CRISPR loci^9^. CRISPR1 and CRISPR2 in *C. sakazakii* had leader sequences and *cas* genes, implying that they were active. As shown in Fig. 2a, the length of consensus repeat sequences in CRISPR1 and CRISPR2 was 29 bp, and their predicted RNA secondary structures were similar to repeat sequence cluster 2, associated with subtype I-E CRISPR-Cas system in *E.coli*^24^.

**Figure 3:**
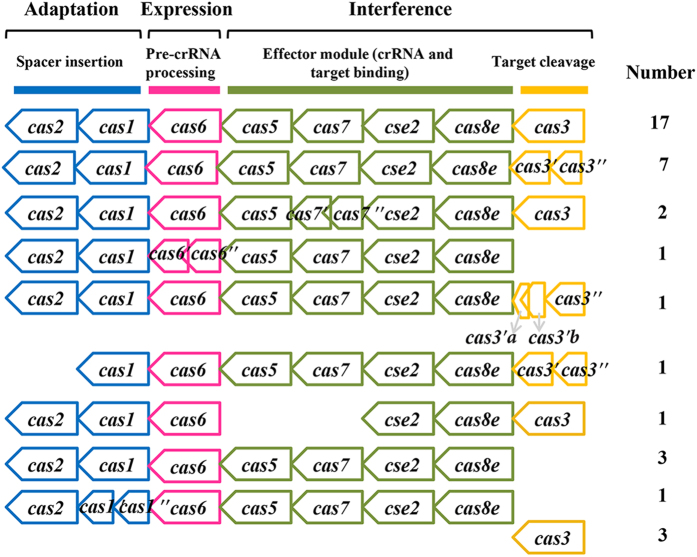
Architecture of genomic loci of CRISPR-Cas systems in *Cronobacter sakazakii.* Gene names follow the current nomenclature and classification. On the left is the arrangement of *cas* gene loci, whereas on the right is the number of strains containing the type of *cas* gene cluster. The *cas* genes are colored according to their different processes.

As shown in Fig. 3, there were total 8 successive co-oriented *cas* genes: *cas2, cas1, cas6, cas5, cas7, cse2, cas8e* and *cas3*. In accordance with previous studies^10^,^21^, the CRISPR-Cas of *C. sakazakii* is subtype I-E. 89.19% (33/37) of the strains were found to have two common *cas* genes (*cas1* and *cas2)*, and the he signature *cas* gene of type I CRISPR-Cas system (*cas3*). Eight *cas3* genes split into helicase *cas3′* and HD nuclease *cas3′′* genes. These split *cas*3 genes were also seen in subtype I-A CRISPR-Cas system^10^. One *cas3′* gene further split into two small compartments (designated as *cas3′*a and *cas3′*b). The split genes were also seen in other *cas* genes (Fig. 3). Whether these genes can translate into active proteins is unknown. Three strains had single *cas3* genes. Additionally, 91.89% (34/37) of the strains had both *cas8e* and *cse2* genes, which encode for signature proteins of CRISPR-associated complex for antiviral defense (Cascade) effector complex of subtype I-E CRISPR-Cas system^10^. A ‘complete’ *cas* locus encompasses at least the full complement of genes for the main components of the interference module^10^. Whether these subtype I-E CRISPR-Cas system variants had activity to protect *C. sakazakii* strains away from phages attack are unknown.

### The correlation of phage infection and spacer presence in CRISPR-Cas systems of *C. sakazakii*

All predicted prophages (n = 131) sequences in *C. sakazakii* strains were extracted to build a local database. To evaluate the protection by CRISPR-Cas system against the foreigner nucleic sequences, we collected all spacers from the five CRISPR arrays to seek the similarity sequences from prophages and plasmids in ACLAME database and our local database in this study. All the spacers with matched protospacers in phages and plasmids belonged to CRISPR1 and CRISPR2, supporting our previous suggestion that CRISPR1 and CRISPR2 were active loci. In this study, we found the entire subtype I-E CRISPR-Cas system like *E.coli* and two active CRISPR arrays in *C. sakazakii* strains. Meantime, there were some spacers matched with phages in the CRISPR arrays, so we speculated that CRISPR-Cas system of *C. sakazakii* could provide defense from phage attacks through immunological memory saved in spacers.

**Figure 4:**
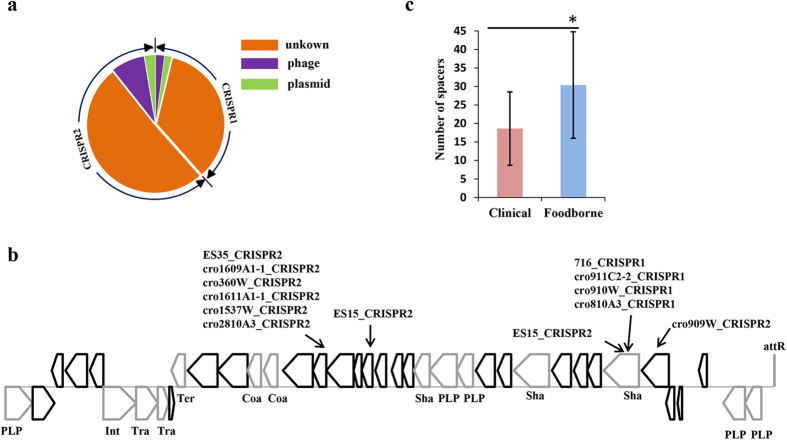
Potential protection provided by CRISPR-Cas systems against phages. (**A**) The pie chart shows the number of spacers from CRISPR1 and CRISPR2 with potential protospacer matches in phage and plasmid. (**B**) Matches of CRISPR spacers with the intact prophage in *C. sakazakii* ES15 strain (location: 1767028-1797368). The sequence regions of prophage matched with the spacers in CRISPR-Cas system are indicated by an arrow. The matched spacer was named based on the name of the isolated strain and the type of CRISPR locus. PLP: phage-like protein; Int: integrase; Tra: transposase; Ter: terminase; Coa: coat protein; Sha: tail shaft. All other genes on the black line encode hypothetical proteins. (**C**) Number of spacers from two active CRISPR arrays in *C. sakazakii* clinical strains and foodborne strains. The strains were colored the same as in Fig.1A. The average number of spacers in clinical strains was less than that of foodborne strains, and the difference had statistically significance. *P < *0.01* (t-test, two tail).

As shown in Fig. 4A, only 11.47% (125/1090) of the spacer sequences in CRISPR1 and CRISPR2 were found to match sequences from phages and plasmids. It may not be exclude the reason for the lack of identified prophages and plasmids in public database. Of the matched sequences, 71.20% (89/125) were phages, suggesting that phages were an important type of HGT in *C. sakazakii*. Moreover, CRISPR2 had a higher proportion of spacers targeted phages (77.08%, 74/96) than plasmids (22.92%, 22/96). The spacers in CRISPR1 did not show the difference. Whether there is a priority to select CRISPR2 loci against phages need to be determined in the future.

20.8% (26/125) of predicted prophages in *C. sakazakii* strains have found protospacers targeted by spacers from CRISPR1 and CRISPR2. As shown in Fig. 4B, one intact prophage from *C. sakazakii* ES15 strain (location: 1767028–1797368) was targeted by spacers, from its host strain and other strains, at five different sequence regions. From the targeted regions, two are in phage tail shaft protein, others in hypothetical non-structural protein. Four strains detected by the matched spacers were infected with the homologous phage. Especially, two different spacers targeting a sequence of prophage were integrated into the CRISPR2 of ES15 strain to enhance its immunological memory, to strengthen the defense in the future. Eight spacer sequences that matched this prophage were detected in strains without this prophage. This supported the idea that CRISPR-Cas system of *C. sakazakii* could provide defense from phage attacks through immunological memory saved in spacers. The number of spacers from two active CRISPRs in clinical strains was significantly less than that of foodborne strains (Fig. 4c). This result may explain the reason for the difference of the average number of prophages between clinical strains and foodborne strains. Touchon *et al*. reported a negative association between the number of spacers in CRISPR arrays and the number of prophages in lysogens^25^. However, there was no significant negative correlation between the number of spacers in CRISPR arrays and prophage frequencies in *C. sakazakii* (Supplemental Fig. 1). The reason for this inconsistency might be that the CRISPR-Cas system does not simply function as a defense system in *C. sakazakii* or that there are other efficient mechanisms against phages. All these speculations need to be determined in the future.

In conclusion, the universal infection of temperate phages is a major factor contributing to genetic diversity and pathogenicity of *C. sakazakii*. A solo class 1, subtype I-E CRISPR-Cas system and five types of CRISPR arrays were found in *C. sakazakii*. CRISPR1 and CRISPR2 loci with high variable spacers were active and showed potential protection against specific phage attacks through immunological memory saved in spacers. At the same time, we found that the number of spacers from active CRISPR loci in clinical strains is significant less than that of foodborne strains, it maybe a reason why clinical strains were found to have more prophages than foodborne strains. More prophages and their related genes may be benefit for the survival and pathogenicity of clinical strains. The rapid gain/loss of prophages and spacers in CRISPR loci is likely to drive the quick evolution of *C. sakazakii*. Our study is an important step towards better understanding the co-evolution of phages and *C. sakazakii*, and presents the importance of further research aimed at deciphering the mechanisms of prophages and CRISPR-Cas systems that affect the pathogenicity and environmental adaption of *C. sakazakii.*

## Methods

### Bacterial strains, genome sequencing, and pan genome analysis

17 *C. sakazakii* strains, isolated from several types of food in China, were selected (Supplemental Table S1). 13 of them belonged to three major sequence types (ST4, ST1 and ST8) of *C. sakazakii* strains in China, three were our newly discovered sequence types (ST266, ST283, ST287), one were ST64^1^. The genomes of *C. sakazakii* strains were fragmented using NEBNext dsDNA Fragmentase (NEB, USA) and Bsp143I (Sau3AI; Fermentas, Lithuania). DNA fragments (500 bp) were purified for preparation of a sequencing library, using QIAGEN GeneRead Library Prep (I) kit (Qiagen, Germany). Samples were subjected to 2 × 250 bp paired-end sequencing, using the Illumina Hiseq 2500 instrument, to generate 1 million reads with 100-fold coverage. The raw data for each bacterium were error-corrected and assembled using SPAdes 3.6.2^26^. The final assemblies were filtered to contain ≥200 bp contigs. Genome annotation was performed using Prokka 1.11^27^. Pan genome analysis was performed by GET_HOMOLOGUES software^28^, using the standard described by Tettelin and collaborators^29^.

### Identification of prophages and CRISPR-Cas system

The whole genome sequences of 17 strains achieved from our study and 42 strains available in NCBI genome database (the strains had more than one submitted genome sequences, only the sequence with fewer scaffolds was selected) were extracted as our sequence set. For many public clinical sequences were from a neonatal intensive care unit outbreak in France 1994, they were multiple isolates from the same strains, then we selected the reference strains as represent according to the primary paper for our analyses (detail in Supplemental Table S2)^30^,^31^. The prophages were identified using PHASTER^32^. We removed prophages with a large number of insertion sequences (IS; >25% of the predicted ORFs). Microsoft Excel was used for all the statistical analysis.

CRISPR array can be detected by CRISPRFinder^33^. CRISPR arrays with two or more identical spacer lengths were identified. The graphical representations (logos) of the patterns in the alignments of all the consensus repeats of specific CRISPR array in *C. sakazakii* were visualized with WebLogo 3.0^34^. Secondary structure prediction of the most frequently occurring repeat sequence was performed using Mfold^35^. We extracted 500 bp, downstream and upstream of the CRISPR array, to find the AT-rich leader region. For *cas* genes, 394 profiles were used for PSI-BLAST analysis of the potential *cas* genes in genomes of *C. sakazakii* strains with e-value 10^–6^, as described previously^10^. The potential *cas* genes related to CRISPR array were identified, and their conserved domain was analyzed using the Conserved Domain Database^36^.

### Determining spacer matches

We extracted all sequences of predicted prophages (n = 131) from *C. sakazakii* strains to build a local database. The similarity search of identified spacer sequences was performed by using BLAST with ACLAME database^37^ and local database in this study. We considered matches to be not less than 84% (minimum of 27/32 matching nucleotides).

### Nucleotide sequence accession numbers

The GenBank accession numbers of the 17 whole genome sequences of *C. sakazakii* strains reported in this article are provided in Supplemental Table S1.

## Additional Information

**How to cite this article**: Zeng, H. *et al*. The driving force of prophages and CRISPR-Cas system in the evolution of *Cronobacter sakazakii. Sci. Rep.* **7**, 40206; doi: 10.1038/srep40206 (2017).

**Publisher's note:** Springer Nature remains neutral with regard to jurisdictional claims in published maps and institutional affiliations.

## Supplementary Material

Supplementary Information
