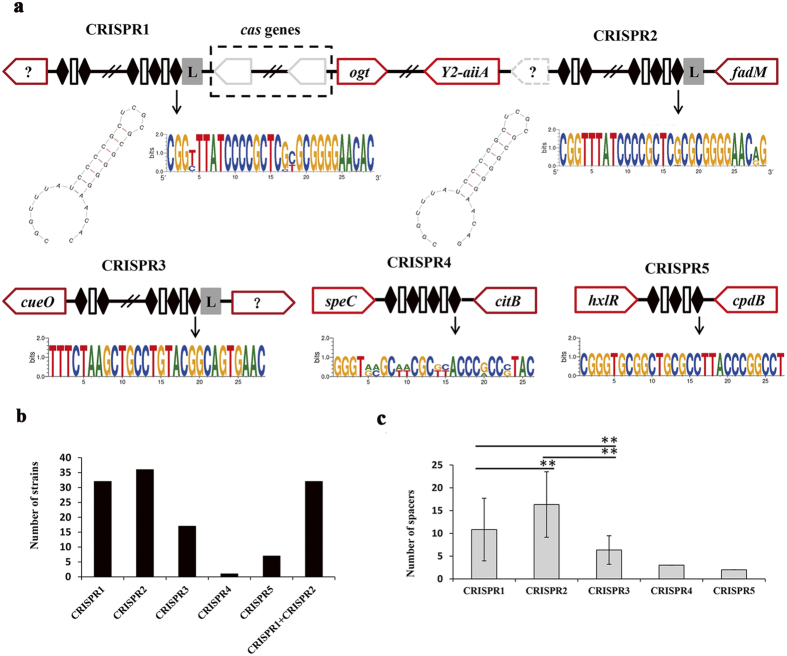# Corrigendum: The driving force of prophages and CRISPR-Cas system in the evolution of *Cronobacter sakazakii*

**DOI:** 10.1038/srep46783

**Published:** 2017-04-26

**Authors:** Haiyan Zeng, Jumei Zhang, Chensi Li, Tengfei Xie, Na Ling, Qingping Wu, Yingwang Ye

Scientific Reports 7: Article number: 40206; 10.1038/srep40206 published online: 01 06 2017; updated: 04 26 2017.

This Article contains an error in Figure 2, where the translation orientation of the hypothetical gene adjacent to CRISPR3 is reversed. In addition, the location of citB and speC beside CRISPR4, along with the sequence logo of CRISPR4 repeats should be reversed. The correct Figure 2 appears below as Figure 1.

## Figures and Tables

**Figure 1:**