# Supplementary material for: The driving force of prophages and CRISPR-Cas system in the evolution of Cronobacter sakazakii
Source: Sci Rep. 2017 Jan 6;7:40206. doi: 10.1038/srep40206 (PMC5216340; doi:10.1038/srep40206)
Supplement: Supplementary Information [file srep40206-s1.pdf]

The driving force of prophages and CRISPR-Cas system in the evolution of *Cronobacter sakazakii*

Haiyan Zeng[1#], Jumei Zhang[1#], Chensi Li[1], Tengfei Xie[1], Na Ling[1], Qingping Wu[1*], Yingwang Ye[1].

[1] State Key Laboratory of Applied Microbiology, South China, Guangdong Provincial Key Laboratory of Microbiology Culture Collection and Application, Guangdong Open Laboratory of Applied Microbiology, Guangdong Institute of Microbiology, Guangzhou 510070, China.

*****Address correspondence to Wu Qingping, wuqp203@163.com

# Authors contributed equally to this work.

Supplemental Table S1. Detailed information and GenBank accession numbers of 17 *Cronobacter sakazakii* strains isolated from food in China.

Strains	Sources	Date	City	ST	N50	GC percent	Genome size	Accession
cro1212W	cold noodles in sauce samples	2012	Xi'an	266	37395	57.50	4.258540	MBSA00000000
cro711B3	deli meat samples	2012	Fuzhou	4	16556	57.19	4.356495	MBRT00000000
cro560W	cold noodles in sauce samples	2012	Sanya	4	30174	57.22	4.522159	MBRU00000000
cro1160W	cold noodles in sauce samples	2012	Harbin	287	32676	57.18	4.574010	MBRY00000000
cro360A2	cold noodles in sauce samples	2012	Zhanjiang	4	154019	56.90	4.732622	MBRP00000000
cro360W	cold noodles in sauce samples	2012	Zhanjiang	8	33358	57.22	4.326341	MBRQ00000000
cro911C2-2	deli meat samples	2012	Nanchang	1	492803	56.67	4.568860	MBRZ00000000
cro810A3	cold noodles in sauce samples	2012	Nanchang	1	22815	57.39	4.333719	MBRR00000000
cro910W	cold noodles in sauce samples	2012	Nanchang	1	51856	57.32	4.465138	MBRS00000000
cro909W	cold vegetables in sauce dishes samples	2012	Nanchang	283	12004	57.70	4.164128	MBRV00000000
cro1611A1-1	deli meat samples	2013	Guangzhou	8	11826	57.58	4.108599	MBRW00000000
cro1509B2	fried rice or noodles samples	2013	Guangzhou	4	101815	57.13	4.567728	MBRO00000000
cro1537W	deli meat samples	2013	Guangzhou	8	72131	57.20	4.541371	MBRX00000000
cro1609A1-1	cold vegetables in sauce dishes samples	2013	Guangzhou	8	20454	57.44	4.249981	MBRN00000000
cro2811A3-1	deli meat samples	2015	Changsha	4	22959	57.50	4.268604	MBRM00000000
cro2810A3	cold noodles in sauce samples	2015	Changsha	8	21825	57.26	4.398006	MBSB00000000
cro2819A3	mushroom	2015	Changsha	64	85999	57.19	4.372502	MBSC00000000

Supplemental Table S2. Information of 42 public *Cronobacter sakazakii* strains used in this study.

Strains	Sources	Country	ST	Date	Genome size
SP291*	environment	Ireland	/	/	4.51878
NM1240*	clinical	USA	/	2008	4.4929
NCTC 8155*	milk	United Kingdom	/	1980	4.61057
NCIMB8272*	milk	United Kingdom	4	1950	4.57948
NBRC102416*	clinical	/	/	/	4.548307
HPB5174*	environment	Ireland	40	2008	4.44461
ES713*	clinical	USA	4	/	4.55101
ES35*	clinical	Israel	/	/	4.34771
ES15*	grain	South Korea	/	/	4.26867
E899*	clinical	France	4	/	3.95731
E764*	clinical	Czech Republic	/	/	4.44086
BAA894*	formula	USA	1	2001	4.53078
ATCC 29544*	clinical	USA	8	1970	4.66357
8399*	clinical	Israel	109	2000	4.66026
2151*	clinical	USA	/	/	4.38275
680*	clinical	USA	8	1994	4.36276
716*	Environment	France	14	1994	4.79652
699*	clinical	France	12	1994	4.66782
703	clinical	France	12	1994	4.61893
708	clinical	France	12	1994	4.66553
696	clinical	France	12	1994	4.66149
690	clinical	France	12	1994	4.66789
700*	clinical	France	13	1994	4.64226
715	formula	France	13	1994	4.55523
714	formula	France	13	1994	4.5523
713	formula	France	13	1994	4.5498
693	clinical	France	13	1994	4.55513
701*	clinical	France	4	1994	4.54745
712	formula	France	4	1994	4.4162
767	clinical	France	4	1994	4.51435
730	clinical	France	4	1994	4.54336
711	clinical	France	4	1994	4.57231
709	clinical	France	4	1994	4.54311
707	clinical	France	4	1994	4.54372
706	clinical	France	4	1994	4.55966
705	clinical	France	4	1994	4.54341
702	clinical	France	4	1994	4.56418
698	clinical	France	4	1994	4.53845
695	clinical	France	4	1994	4.54632
694	clinical	France	4	1994	4.538
692	clinical	France	4	1994	4.53851
691	clinical	France	4	1994	4.53866

*means the genome sequences of the strains which were selected for our analyses.

26 strains isolated in France during 1994 were from a neonatal intensive care unit outbreak, we selected clinical strains 699, 700 and 701 to represent other isolates from the same strains.

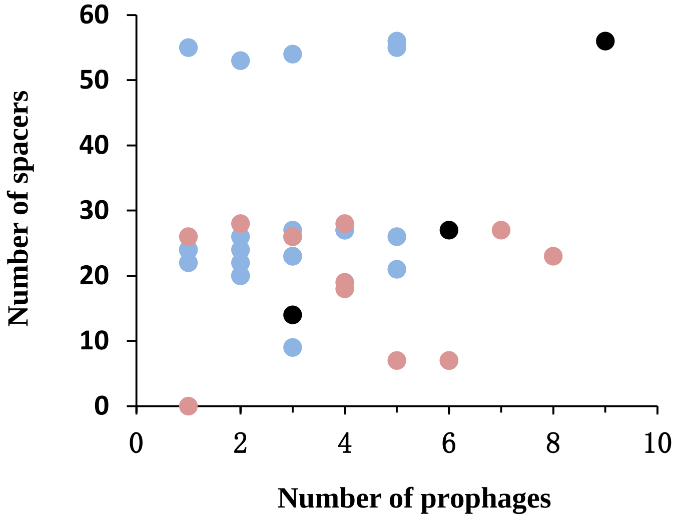

Supplemental Fig.S1. Relation between total number of spacers from two active CRISPR loci and the number of prophages in *Cronobacter sakazakii*.

There was no significant correlation between the number of spacers in CRISPR arrays and prophage frequencies in *C. sakazakii*. The strains were colored the same as in Fig.1A.
